# Predictable Variation of Range-Sizes across an Extreme Environmental Gradient in a Lizard Adaptive Radiation: Evolutionary and Ecological Inferences

**DOI:** 10.1371/journal.pone.0028942

**Published:** 2011-12-14

**Authors:** Daniel Pincheira-Donoso

**Affiliations:** Centre for Ecology and Conservation, College of Life & Environmental Sciences, University of Exeter, Streatham Campus, Exeter, Devon, United Kingdom; Centre National de la Recherche Scientifique, France

## Abstract

Large-scale patterns of current species geographic range-size variation reflect historical dynamics of dispersal and provide insights into future consequences under changing environments. Evidence suggests that climate warming exerts major damage on high latitude and elevation organisms, where changes are more severe and available space to disperse tracking historical niches is more limited. Species with longer generations (slower adaptive responses), such as vertebrates, and with restricted distributions (lower genetic diversity, higher inbreeding) in these environments are expected to be particularly threatened by warming crises. However, a well-known macroecological generalization (Rapoport's rule) predicts that species range-sizes increase with increasing latitude-elevation, thus counterbalancing the impact of climate change. Here, I investigate geographic range-size variation across an extreme environmental gradient and as a function of body size, in the prominent *Liolaemus* lizard adaptive radiation. Conventional and phylogenetic analyses revealed that latitudinal (but not elevational) ranges significantly decrease with increasing latitude-elevation, while body size was unrelated to range-size. Evolutionarily, these results are insightful as they suggest a link between spatial environmental gradients and range-size evolution. However, ecologically, these results suggest that *Liolaemus* might be increasingly threatened if, as predicted by theory, ranges retract and contract continuously under persisting climate warming, potentially increasing extinction risks at high latitudes and elevations.

## Introduction

The dynamics of species geographic range-size evolution are mediated by ecological, physiological and physical factors that set the boundaries for viable dispersal [1], [2], [3]. As a result, most species have ranges restricted to particular areas of the planet, and most are restricted to particular environmental spots where even local habitat fragmentations prevent dynamic migration between them [4]. Multiple hypotheses have attempted to elucidate the causes, and hence the predictability, of current patterns of range-sizes in nature under the context of different organismal and environmental factors [5]. However, despite decades of research, the search for general explanations underlying range-size variation remains a challenging endeavour [1], [5].

Research on the ecological and evolutionary dynamics of range limits has become increasingly important with the observation that species distributions are rapidly altered by human-induced climate change. In recent years, numerous reports have shown ongoing range alterations across diverse organisms consistent with climate change predictions [1], [3], [6], [7], [8], [9], [10], [11], [12], [13]. For example, climate change-driven range alterations have been shown in groups as diverse as butterflies [9], [13], frogs [14], [15], and birds [16]. As predicted, these range alterations have been involved in population declines or in actual extinctions in species where adaptive responses to environmental changes or dispersal into new areas have been obstructed by genetic or physical barriers [6], [8], [9], [10], [17], [18]. The potential for threatened species to escape extinction following rapid climatic shifts depends on multiple biological features. For example, rapid adaptive responses to climate change are more likely in species with short generation times, such as insects [19], [20], but appear less likely in longer-generation organisms, such as vertebrates [17], [21], [22]. Also, species distributed at high latitudes and elevations are expected to experience threats as range alterations caused by upward and poleward advances of warming climate may cause range contractions while available space to disperse tracking historical niches progressively declines, such as on mountaintops [8],[10],[12],[23],[24],[25]. Subsequently, range contractions and fragmentations may compromise population persistence via reduced genetic diversity [4]. Warming will also promote dispersal of species from warm areas that may compete with resident species from historically cold areas, intensifying environmental stress, population damage, and extinction ([6], [22], [23], but see [26]). Therefore, species with longer generations from high latitudes and elevations and with restricted range-sizes are expected to become particularly threatened under persisting climate warming.

In this context, the macroecological study of spatial variation of geographic range-sizes across environmental gradients is of primary interest to infer factors involved in the evolution of their boundaries, and hence, to reinforce predictions about potential large-scale responses to changing climates. Particularly, although cold climate species with restricted ranges are expected to be more threatened by climate warming, a widely known macroecological generalization (known as Rapoport's rule) posits that species range-sizes tend to increase with decreasing climatic temperatures along biogeographical gradients [27], [28], [29]. Therefore, in lineages where this trend holds, larger ranges toward higher latitudes-elevations may contribute to counterbalance the impact of climate warming, potentially retarding range contractions and extinctions. However, empirical support to this rule is equivocal, being increasingly discredited as a generality [29].

The use of prominent adaptive radiations offers excellent conditions to investigate within the phylogenetic boundaries of a given lineage (where evolutionary events are related and comparable) the impact of factors expected to affect the trajectories of ecological and evolutionary processes, such as range-size variation. Here, I investigate the questions whether range-sizes among species of the prominent *Liolaemus* (family Liolaemidae) lizard adaptive radiation vary predictably across one of the most extreme environmental gradients known for a single lizard genus, and whether this variation would most likely enhance or counterbalance potential threats under persistent climate change (i.e. whether range-sizes increase or decrease with latitude-elevation). In addition, I investigate whether range-sizes are influenced by interspecific differences in body size, as suggested by previous studies. However, the direction of these relationships is inconsistent. While some studies reveal that larger ranges result from higher ecological tolerance and competitiveness of larger species [1], [30], [31], [32], [33], [34], others show both positive or negative covariations [35], or even triangular relationships [1]. The *Liolaemus* radiation provides an ideal model organism to address these questions. Consisting of 220+ species, these lizards have extensively radiated throughout central and southern South America and have colonized a unique variety of environments [36], occurring from the extreme Desert of Atacama to Patagonian areas that include the southernmost place inhabited by lizards, and from sea level to over 5000 m of elevation [37], [38], [39], [40]. Across this environmental gradient, *Liolaemus* species have evolved a diversity of range-sizes, life histories and thermal adaptations [38], [41], [42], [43], [44] that offer the ideal evolutionary scenario to conduct large-scale comparative analyses within a single radiation.

## Results

**Figure 1:**
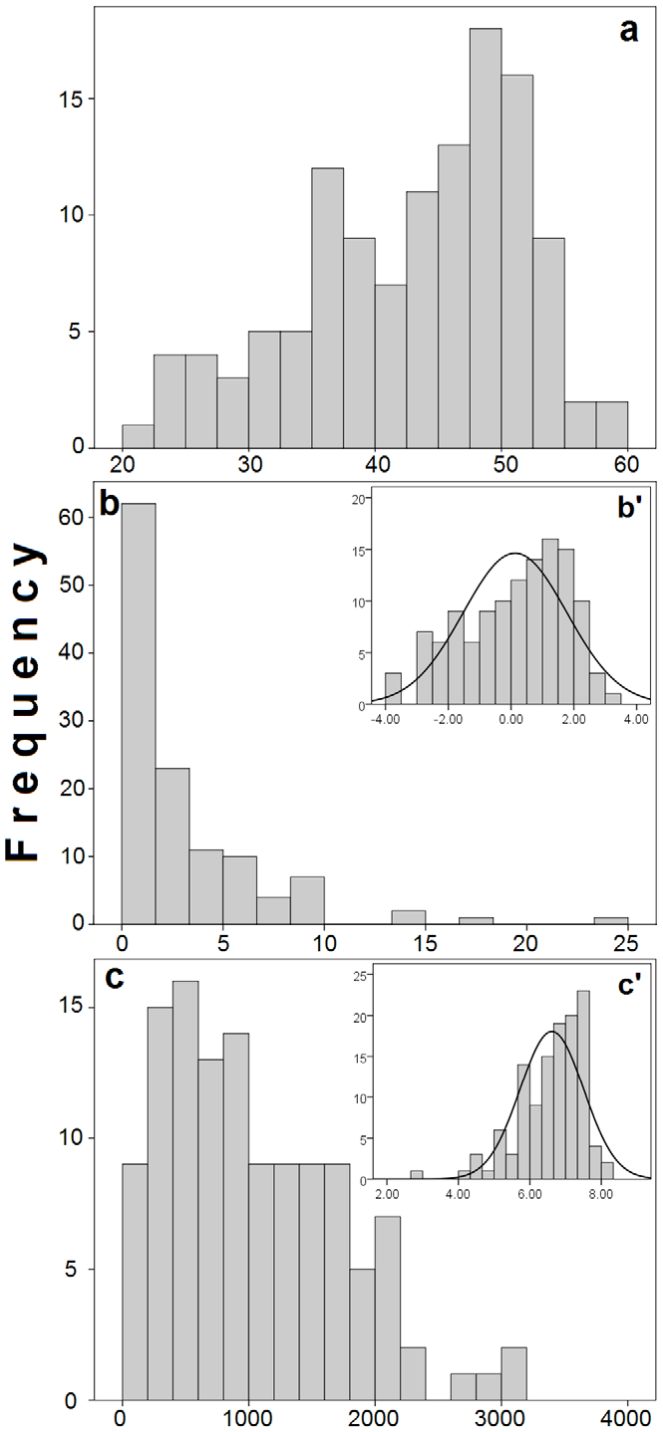
Frequency distributions of geographic locations of *Liolaemus* species expressed as a combination of latitude and elevation under the adjusted latitudinal midpoint (ALM, a), and of their latitudinal (expressed in degrees of latitude, b) and elevational (expressed in metres of elevation, c) geographical range sizes expressed in arithmetic scales, and in their corresponding logarithmic scales (b' for latitude, c' for elevation).

The range-size frequency-distributions on arithmetic scales in *Liolaemus* are consistently right-skewed in both latitudinal and elevational ranges (Kolmogorov-Smirnov test, latitudinal range: *D*(121) = 0.22, *P*<0.001; elevational range: *D*(121) = 0.11, *P* = 0.002; Fig. 1b, c), and hence, the tendency within the genus is towards geographically restricted species, with some examples of extreme historical dispersal ability. In contrast, the frequency-distribution of the ALM is left-skewed (Fig. 1a). As shown by these frequency-distribution plots, both latitudinal (range mean = 02°58′S±3.8 SD, range = 0°01′S–23°33′S, mode = 0°06′S) and elevational ranges (range mean = 1011±691 m, range = 20–3153 m, mode = 300 m) show considerable interspecific variation. Logarithmic transformations (ln) of arithmetic frequency distributions of latitudinal and elevational ranges reduced skewness, but failed to reach normality (latitudinal range: *D*(121) = 0.12, *P*<0.001; elevational range: *D*(121) = 0.09, *P* = 0.01; Fig. 1b', c').

**Figure 2:**
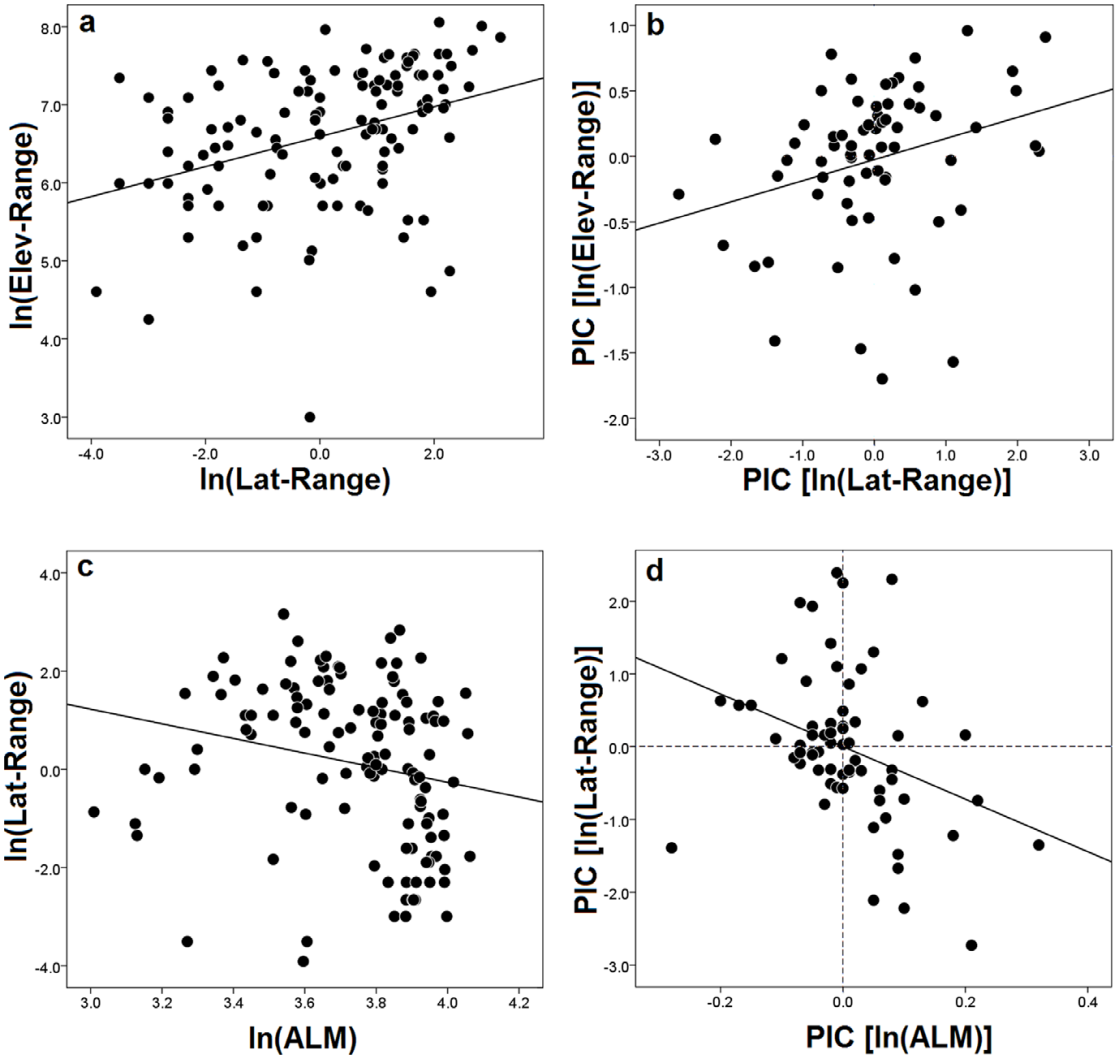
Analyses of range size variation in *Liolaemus* lizards, showing correlations between latitudinal and elevational ranges based on conventional (a) and phylogenetic analyses (b), and regression analyses of latitudinal range variation as a function of adjusted latitudinal midpoint (ALM) in both raw (c) and phylogenetically controlled data (d). Abbreviations include latitude (Lat), elevation (Elev), and phylogenetic independent contrasts (PIC).

Quantitative analyses of range-size variation revealed qualitatively identical results when employing both conventional statistics and phylogenetic comparative methods (Table 1), which suggests a substantial consistency between predictors and range-size independent of the analytical approach employed. This finding contrasts with a previous similar study in *Liolaemus* lizards based on a smaller sample [42] where results from both conventional and phylogenetic analyses differed significantly. The test of the primary question whether range-sizes vary predictably across an environmental and geographical gradient revealed that latitudinal range-sizes decrease predictably with increasing latitude-elevation (ALM), and hence, with decreasing climatic temperatures (Table 1; Fig. 2c,d). Also, figure 2c shows that the magnitude of residuals below the fit line is greater than above it, and hence, latitudinal range-sizes deviate more strongly towards smaller ranges than expected than the deviations towards larger ranges than expected. However, a weak, non-significant, relationship was detected between ALM and elevational range-size (Table 1; figure not shown), despite the significant positive correlation between latitudinal and elevational range-sizes (Table 1; Fig. 2a,b). Analyses involving body size showed that the historical dispersal ability of *Liolaemus* species appears to be unrelated to average species size, as no predictable covariation was observed (Table 1). When differences in body size between the sexes were accounted for, similar relationships were observed between body size and range-size variation (Table 1). These results are also entirely consistent between conventional and phylogenetic analyses (Table 1).

**Table 1:** Conventional (non-phylogenetic, abbreviated as NP) and phylogenetic (based on phylogenetic independent contrasts, abbreviated as PIC) analyses of large-scale patterns of latitudinal (Lat) and elevational (Elev) range size variation as a function of geographical distribution (adjusted latitudinal midpoint, ALM) and body size (SVL; for two of these tests the effect of sexual size dimorphism, SSD, is controlled for) in the lizard genus *Liolaemus*.

Analysis	Test	*N*	*r*	*R^2^*	*F (df)*	*P*
Range (Elev) on Range (Lat)	NP	121	0.36	–	–	***<0.001***
	PIC	68	0.29	–	–	***0.02***
Range (Lat) on ALM	NP	121	−0.21	0.04	5.42 (1,119)	***0.02***
	PIC	68	−0.35	0.12	9.02 (1,66)	***<0.01***
Range (Elev) on ALM	NP	121	0.15	0.02	2.80 (1,119)	0.1
	PIC	68	0.17	0.03	1.97 (1,66)	0.17
Range (Lat) on SVL	NP	115	−0.04	0.002	0.18 (1,113)	0.67
	PIC	65	0.05	0.002	0.13 (1,63)	0.72
Range (Elev) on SVL	NP	115	0.08	0.01	0.68 (1,113)	0.41
	PIC	65	0.2	0.04	2.57(1,63)	0.11
Range (Lat) on SVL (SSD)	NP	115	−0.07	0.01	0.31 (2,112)	0.74
	PIC	65	0.2	0.04	1.13 (2,62)	0.33
Range (Elev) on SVL (SSD)	NP	115	0.08	0.01	0.34 (2,112)	0.71
	PIC	65	0.2	0.04	1.27 (2,62)	0.29

Relationships between latitudinal and elevational ranges are analysed using correlations as no causal weight can be attributed to any of these variables. See methods section for additional details.

## Discussion

This study provides evidence that latitudinal range-sizes in *Liolaemus* lizards decrease predictably with increasing latitude-elevation across an extreme environmental gradient. Hence, these observations entirely reverse the pattern predicted by Rapoport's rule [28], [29], while no effects of distribution were observed on elevational range-sizes. The phylogenetic analyses revealed the same relationship. These results contrast with a previous study on a smaller sample of *Liolaemus* species, where non-historical analyses revealed a positive relationship between latitudinal range-size and species latitudinal and elevational distributions, while phylogenetic tests showed no association between these variables [42]. In addition, I found that body size appears not to influence range-size variation in these lizards. Collectively, these results suggest an historical connection between the radiation of *Liolaemus* lizards into cold-climate environments and their dispersal potential, and that the evolutionary outcome of decreasing ranges with increasing latitude-elevation may result in higher levels of population vulnerability and potentially extinction in colder climate species, as a result of range contractions if upward and poleward climate warming persists.

### Evolutionary inference and ecological expectations of range-size dynamics

The observed relationships between range-size and environmental gradients can be interpreted from, first, an evolutionary perspective, and second, an ecological perspective involving potential consequences of climate warming. Evolutionarily, these results suggest that the historical dynamics of latitudinal range limits have been influenced significantly by the environmental conditions encountered by *Liolaemus* during their radiations into high latitudes and elevations, where the increasingly colder and unstable climatic conditions stand as primary candidate factors. However, given that only latitudinal ranges predictably decrease as a function of increasing ALM, in contrast to elevational ranges (Table 1), range limits are unlikely to be restricted by thermophysiological demands of colder climates alone. This inference is supported by a previous study where thermal tolerance was shown to increase with increasing latitudes-elevations across *Liolaemus* species [42], in agreement with theory [45] and additional empirical evidence coming from other ectotherms [46], [47]. If range dispersal relied exclusively on thermophysiology, it would be expected that given greater thermal tolerance in colder climates, latitudinal ranges would not be restricted by declining climatic temperatures (as observed in elevational ranges), in contrast to the results of this paper. Therefore, this suggests that additional factors associated with higher ALMs play an important role in shaping range-size variation in these lizards (e.g., [1], [48]). In the case of cold climate *Liolaemus* species, range boundaries are known to be influenced by the irregular topography of the Andes, characterized by multiple mountain peaks spread across thousands of kilometres of latitude, and where an important part of this evolutionary radiation has taken place [38], [43], [49]. This topographical scenario then imposes severe physical barriers for latitudinal dispersal, while elevational dispersal would not be equally restricted within mountains, which would be further facilitated by greater thermal tolerance. However, the fact that latitudinal ranges are affected by Andean topography necessarily indicates that elevational dispersal is possible only within certain limits. Otherwise, there would be no limits to latitudinal distribution. Indeed, several Andean *Liolaemus* species are restricted to ‘elevational islands’ (as observed in other mountain lizards; e.g. [22]) represented by high elevation zones isolated by lower elevation valleys and cliffs from similar high elevation peaks where related species occur (e.g., [50], [51]). Therefore, species dispersal between high elevation areas through lower elevation corridors appears, in fact, to be impeded.

Despite greater thermal tolerance of cold-climate *Liolaemus*, mountain restrictions may be explained by at least three evolutionary scenarios that potentially apply for cold-climate lizards in general. First, the evolution of increasing thermal tolerance in colder climate species might only be possible within a narrow range (e.g., [41]), allowing elevational dispersal within similarly narrow thermal limits. Second, dispersal can be impeded, independent of thermal selection, if phylogenetic niche conservatism precludes lizard emigrations from elevationally restricted environmental patches even in the absence of geographical barriers for range expansion (e.g., [2]). For example, isolated vegetational areas determined by climatic conditions [52], [53] associated to particular geological formations, such as rocky outcrops, sustain different *Liolaemus* species and assemblages in different areas of the Andes (see also [22], [51], [54]). Third, independent of climatic constraints on thermoregulation for ecological and reproductive activities, the evolution of viviparity in cold climates can hamper lizard dispersal along elevational gradients. The detrimental effects that cold and unstable environments exert on externally incubating eggs has forced cold climate lizards in general [55], including *Liolaemus* [43], to evolve viviparity. Given that viviparity is tremendously costly in warm environments, and hence mostly viable only in cold climates [55], and almost entirely impeded to re-evolve into oviparity [55], [56], the evolution of viviparity can be regarded as a major factor precluding expansion of cold climate lizards into warmer environments, such as downward dispersal in mountains to access lower elevation corridors. In accordance with this alternative, almost all known cases of viviparous *Liolaemus* species are restricted to high latitudes-elevations [36], [43].

Although the independent or combined effect of the above factors may provide an explanation for the observed patterns of distributional range variation among Andean *Liolaemus*, it may not fully explain the occurrence of small ranges among several Patagonian species, where climates are cold given the high latitudes, but the Andes decrease considerably in elevation [57]. Therefore, in these cold latitudes, low temperatures are constant across extensive, flat, areas with considerably less topographic complexities and environmental fluctuations compared to the Andes. Yet, as in the Andes, some Patagonian *Liolaemus* are isolated in *mesetas* (trap basalts of up to 1,700 m of elevation), but these mesetas are unlikely to impose severe restrictions for dispersal as a generality. For example, while some Patagonian species are isolated on elevated mesetas (e.g. *L. archeforus* and *L. silvanae*), other species (e.g. *L. lineomaculatus*) are geographically widespread, and coexist in different areas of their distribution with other *Liolaemus* restricted to smaller ranges [39], [58]. However, despite these differences between Andean and Patagonian ecosystems, some of the three scenarios detailed above may at least in part account for the restricted distribution of lizards in Patagonia. For example, rock-specialist *Liolaemus* may be forced to remain in bouldery areas, as observed in *Phymaturus* lizards (sister genus to *Liolaemus*) in Patagonia [59], [60].

On the other hand, ecologically, these results may be of conservation concern as the observed negative relationship between latitudinal range and ALM suggests that cold climate *Liolaemus* may be at higher risk of population decline under persisting climate change via range retractions and contractions [3] and habitat fragmentation [61]. Empirical studies have repeatedly shown that climate warming exerts particularly severe negative impacts on species from high latitude-elevations [4], [7], [8], [24], [25], [62], [63], often characterized by hotspots of high endemism [12], [64]. Indeed, in a number of lineages, range-restricted species from high latitudes-elevations are currently experiencing dramatic fragmentation, range retractions and contractions that translate into rapid extinction rates [4], [8], [10], [15]. *Liolaemus* biodiversity may face increasing extinction risks through different processes linked to persisting climate warming. First, assuming some dispersal ability, as species move upward and poleward tracking their historical niches as a result of warming advances in the same direction, dispersal can be impeded by declines in the quality and quantity of available space [8], [10]. Although upward and poleward range expansions may counterbalance range retractions at the lowest latitudinal and elevational distributional limits (where range retractions take place), dispersal might be particularly hampered in species from high Andean elevations and extreme Patagonian latitudes, where mountaintops and coastlines set absolute limits on dispersal. Second, persisting range retractions are expected to increase habitat fragmentation, thus increasing risks of population declines caused by genetic crises with high fitness costs, for example via increased inbreeding rates, and hence, reduced heterozygosity and greater inbreeding depression [4], [65]. Third, warming advances toward historically cold areas are expected to facilitate invasions of species from warm areas, resulting in increasing intensity of competition through, for example, resource competition or predation [22], [23]. Finally, it has been shown that lizard extinctions may occur in structurally intact habitats when climate warming imposes alterations to thermoregulatory behaviour. Lizards prevent body overheating mostly by intermittent retreats into cooler shelters during hot days [21], [66]. With climate warming, lizards will be forced to spend longer periods retreated in these shelters, resulting in reduced opportunities for reproduction and foraging. Because the breeding season requires significant energy intakes to be allocated in reproduction, lizards experiencing climate warming are expected to suffer severe energetic shortfalls [22]. For viviparous species (see third evolutionary scenario above), mostly restricted to high latitudes-elevations [43], [55], this may incur in even greater fitness costs as the high energy requirements of pregnant females to fully develop embryos are accompanied by considerable foraging risks caused by the detrimental impact of the pregnancy burden on escaping efficiency [55], [67]. These factors are expected to interact in additional ways with some of the three evolutionary scenarios described above to functionally link historical dynamics of dispersal with future consequences under climate change. For example, if niche conservatism is important, habitat fragmentation may become an important factor behind depletion of genetic diversity in *Liolaemus* populations.

Alternatively, species facing climate warming can escape extinction through rapid genetic responses to the changing climate [4], [6], [23]. However, as stated above, rapid adaptations are likely to occur in short-generation organisms [19], but seem less likely in larger organisms like lizards [17], [22]. Therefore, under any of the warming-related scenarios described in the previous paragraph, *Liolaemus* biodiversity from high latitudes-elevations may become increasingly threatened under persisting climate change.

### Body size and range-size variation

The influence of body size on most evolutionary and ecological processes has led to suggest that body size mediates differential dispersal ability among different sized species, and hence, that body size might predict range-size [1], [32], [34], [35]. However, *Liolaemus* body sizes are unrelated with interspecific variation in range-size, and no other pattern (e.g. triangular distributions of data points; see Gaston, 2003) is present. This finding is consistent with a recent study on *Liolaemus* range-size variation as a function of body size [42], and with previous observations that *Liolaemus* body sizes do not vary predictably with latitude-elevation gradients [38], [68], which, in contrast, are related with range-size variation (see above).

Despite the lack of predictable covariation between body size and range-size in *Liolaemus*, it seems unlikely that body size does not influence dispersal ability in these lizards. An important difficulty that may preclude the identification of a common effect of body size is that no general tendencies are always expected to exist as several factors are known to interact in different ways across different phylogenetic groups and environmental contexts. For example, Bowler & Benton [35] suggested that dispersal ability is context-dependent, and that depending on given selective regimes dispersal ability may or may not be related with body size. This is probably the case in *Liolaemus*. Since these lizards inhabit one of the widest environmental ranges known within reptiles, multiple selective contexts may operate across species to shape specific body size-dispersal ability relationships, potentially impeding the detection of a generalized mechanism from a generalized pattern involving the relationship between body size and range dimensions.

## Materials and Methods

### Data collection

Data were collected for a total sample of 121 *Liolaemus* species (Table S1) representing all major clades within the genus and occurring in a latitudinal and elevational range that represents its entire diversity (e.g., [36]). Therefore, this dataset covers the phylogenetic and ecological diversity that has resulted from the evolutionary radiation of this group. Data comprise information on geographical distribution and body size per species.

Geographical data consist of species-level information for spatial location and range-size in latitude and elevation. These data have been derived from a number of published sources [38], [39], [42], [43], [44], [58], [68], [69], [70], [71] and from a total record of ∼8,500 specimens from institutional collections (see Appendix S1; the use of the *Liolaemus* data for publication purposes has specifically being granted by all the listed institutions in this appendix) and field records. First, spatial location of species was estimated using a distributional midpoint approach, where a unique spatial point derived from the distributional data per species is used as a predictor of range-size [29], [72]. Given that this study's question focuses on range-size variation across an environmental gradient, I used the adjusted latitudinal midpoint (ALM) variable as an indicator of species distribution, which integrates in a single scale the climatic variation from decreasing environmental temperature in latitude and elevation [42], [73]. The ALM is calculated based on the assumption that temperatures in elevational transects decrease 0.65°C for each 100 m of increased elevation [42], [73]. To correct the dataset for latitudinal and elevational covariation in temperature, Cruz *et al.* [42] obtained a correction factor, computed for the latitudinal range occupied by *Liolaemus* lizards (based on the above 0.65°C for each 100 m) that consists in adding 1.752° (latitude) for every 200 m increases in elevation on altitudinal midpoint values higher than 699 m above sea level. Thus, Cruz *et al.* [42] derived a corrected latitudinal value for latitude and elevational thermal covariation with the formula *y* = 0.009*x*–6.2627, where *x* represents the altitudinal midpoint for each species, and *y* the corrected temperature for latitude, which is added to the latitudinal midpoint for each species. This results in ALM values for South American areas where *Liolaemus* occur [42]. The ALM scale is intuitively simple to interpret, as increasing ALM values represent the integrated effect of increasing latitude and elevation, and hence, a decrease in environmental temperature. Then, range-size variation was analyzed separately for latitudinal and elevational ranges as a function of ALM, where the minimum and maximum records of latitudinal and elevational distribution per species were taken as the limits of the range. Both variables were selected because they are expected to reflect the magnitude of species tolerance to different climatic and ecological conditions experienced by a single species.

Body size data were obtained from a total sample of 4,554 specimens (Table S1). I used snout-vent length (SVL) as a proxy for body size. SVL is the standard body size measure in lizards as it is simple to measure in living and preserved specimens, and covaries with ecological, life history and morphological traits [74], [75], [76], [77]. Given that lizards continue to grow after sexual maturity, it is difficult to estimate standard body size. Therefore, it has been suggested that intermediate percentiles between the mean body size and the largest recorded specimen (both extensively used) provide better estimates of adult size (e.g., [78]). Hence, SVL was obtained using means from the largest two-thirds of the adult samples (e.g., [38], [79]) to avoid under- or overestimations of body size. For analyses, a single SVL value per species was obtained by averaging male and female SVL averages. This approach is more appropriate than pooling all available adult specimens per species to calculate a single mean, as the average would be influenced by the number of males and females in the sample, and hence, by the overall frequency distribution of body size. I then calculated sexual size dimorphism (SSD) with the formula ln(male size/female size) [80]. SSD was then included in the regression models (as an additional predictor) where body size is the main predictor, in order to account for a potential effect of the magnitude of size differences between the sexes, which are in turn obscured by a single species SVL value.

### Statistical analyses and phylogenetic control

A comparative approach based on species-level data was employed. Prior to statistical analyses, variables were ln-transformed to reduce skew and homogenize variances [81]. To investigate the questions whether range-size variation among species is a function of variation in midpoint geographical location (ALM) and of body size (SVL), regression analyses were performed. Given that trait expressions and environments recorded in closely related species can be influenced by their common phylogenetic history, data points from related species in a clade cannot be regarded as independent values for statistical analyses [82], [83]. Therefore, I conducted the same regressions employing, first, conventional (non-phylogenetic assuming a star phylogeny), and then phylogenetic statistical analyses to account for potential phylogenetic effects and infer correlated evolution between variables. Results from both analyses are reported to evaluate the consistency of expected effects of predictors (ALM and SVL, separately) on the response variable (range-size).

For phylogenetic analyses, I used a *Liolaemus* phylogeny (Fig. S1) containing 68 of the 121 species for which data were available (and which were included in non-phylogenetic analyses). For analyses of body size and range-size, SVL data were missing for three of the species in the phylogeny (Table S1), and hence, these phylogenetic analyses were reduced to 65 species. The phylogeny was derived from two previous phylogenetic hypotheses inferred by Espinoza *et al.* [49] and Abdala [84]. Phylogenetic studies of evolutionary relationships within *Liolaemus* have consistently revealed the existence of a major monophyletic clade nested within the genus, known as *boulengeri* complex (e.g., [43], [49], [84], [85]), which has recently been studied by Abdala [84]. Therefore, I used Espinoza *et al.*'s [49] tree as the basis for the *Liolaemus* phylogeny, but replaced the monophyletic *boulengeri* complex with that of Abdala [84] since this phylogenetic hypothesis contains a larger number of species sampled in my dataset. Since these two phylogenetic trees were inferred using combined molecular and morphological data [49], [84], branch lengths were set equal to 1.0, and a speciational Brownian motion model of evolutionary change was employed for phylogenetic analyses [49], [86], [87]. Then, Felsenstein's standardized phylogenetic independent contrasts (PIC) [82] were calculated from this phylogeny using the software COMPARE version 4.6b [88]. I obtained standardized PIC for all the variables involved in the analyses (given that this approach results in *n*-1 independent contrasts, phylogenetic regressions contain 67 and 64 values for the 68-species and 65-species trees, respectively). With PIC, the degree of covariation between variables reflects the potential (but not causation) for these variables to have been functionally related during evolutionary change (e.g. evolutionary dependence between two traits is inferred if large changes in the contrasts of one variable are paralleled by large changes in the contrasts of the other). Regressions based on PIC were forced through the origin [82], [83], [89].

## Supporting Information

Figure S1 Phylogenetic relationships of *Liolaemus* lizard species inferred from combined molecular and morphological data (according to refs. [49], [84]). See main text for details.
(TIF)

Table S1 Summary of *Liolaemus* species included in this study.
(DOC)

Appendix S1 This appendix contains references to the institutions that kindly provided permission to study their *Liolaemus* collections. Important part of the data used in this study comes from these museum collections (see Materials and methods).
(DOCX)
