# Supplementary figures and images for: Predictable Variation of Range-Sizes across an Extreme Environmental Gradient in a Lizard Adaptive Radiation: Evolutionary and Ecological Inferences
Source: PLoS One. 2011 Dec 14;6(12):e28942. doi: 10.1371/journal.pone.0028942 (PMC3237565; doi:10.1371/journal.pone.0028942)

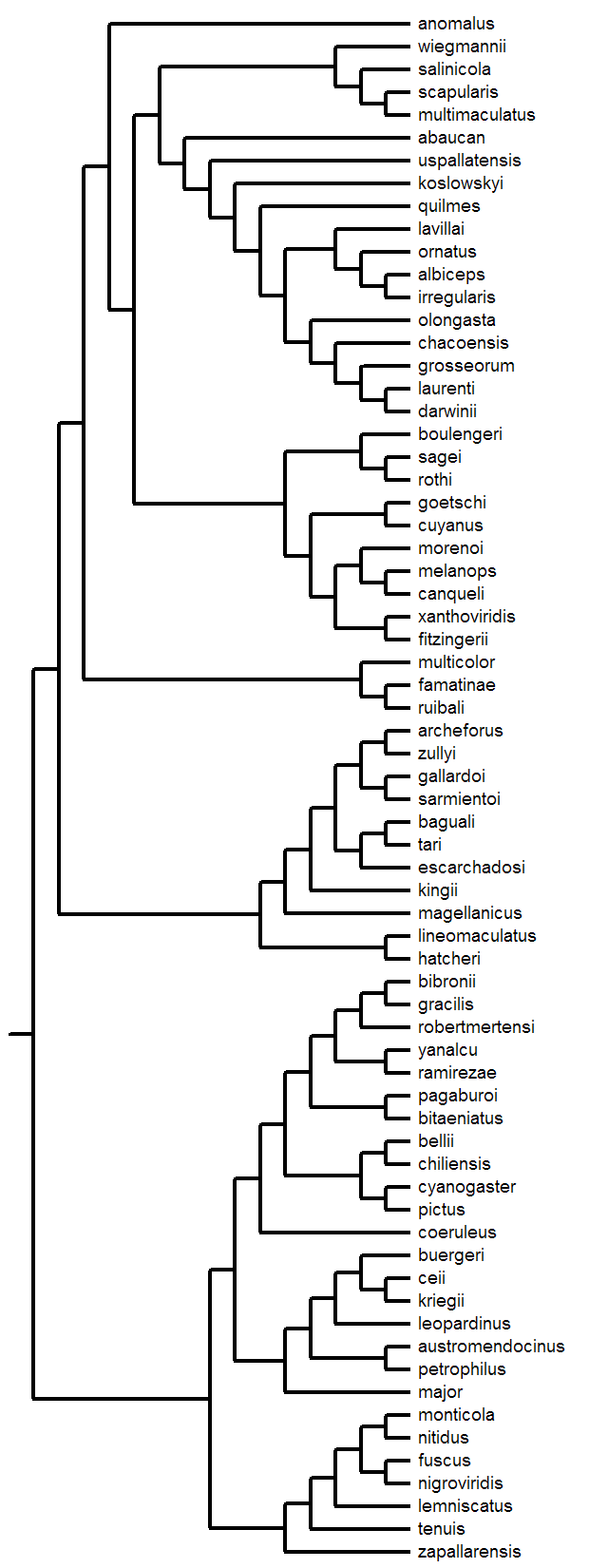

Supplement: Figure S1 — Phylogenetic relationships of Liolaemus lizard species inferred from combined molecular and morphological data (according to refs. [49], [84]). See main text for details. (TIF) [file pone.0028942.s001.tif]
