# Supplementary material for: Predictable Variation of Range-Sizes across an Extreme Environmental Gradient in a Lizard Adaptive Radiation: Evolutionary and Ecological Inferences
Source: PLoS One. 2011 Dec 14;6(12):e28942. doi: 10.1371/journal.pone.0028942 (PMC3237565; doi:10.1371/journal.pone.0028942)
Supplement: Table S1 — Summary of Liolaemus species included in this study. (DOC) [file pone.0028942.s002.doc]

**Table S1.** Summary of *Liolaemus* species included in this study. Species for which phylogenetic information is available are shown in bold. Abbreviations are SVL for snout-vent length, *m* for males and *f* for females. Samples sizes are not provided for species for which size data has been taken from the literature, or from a personal source (details can be found in the original sources, shown at the bottom of the table). Species ordered alphabetically. Sample size (*N*) refers exclusively to the number of specimens used for analyses involving body size, and not for the entire available data used for geographical information. For some species, no body size data were available (indicated), and hence they were removed from those corresponding analyses. Differences in distributional ranges in relation to previous studies (e.g. Pincheira-Donoso et al. 2008a, b) result from new data collected by the author or by new published information.

| **Species** | ***N*** | **SVL** | | **Latitudinal**  **Range** | **Elevational**  **Range** |
| --- | --- | --- | --- | --- | --- |
|  |  | ***m*** | ***f*** |  |  |
| ***L. abaucan***  ***L. albiceps***  *L. alticolor*  ***L. anomalus***  *L. arambarensis*^(1)^  *L. araucaniensis*  ***L. archeforus***  *L. atacamensis*  *L. auditivelatus*  ***L. austromendocinus***  *L. azarai*  ***L. baguali***  ***L. bellii***  ***L. bibronii***  *L. bisignatus*  ***L. bitaeniatus***  ***L. boulengeri***  ***L. buergeri***  ***L. canqueli***  ***L. ceii***  ***L. chacoensis***  *L. chehuachekenk*  ***L. chiliensis***  *L. chillanensis*  ***L. coeruleus***  *L. constanzae*  *L. curicensis*  ***L. cuyanus***  ***L. cyanogaster***  ***L. darwinii***  *L. eleodori*  ***L. escarchadosi***  *L. fabiani*  ***L. famatinae***  *L. fitzgeraldi*  ***L. fitzingerii***  *L. foxi*  ***L. fuscus***  ***L. gallardoi***  ***L. goetschi***  ***L. gracilis***  *L. gravenhorstii*  ***L. grosseorum***  *L. gununakuna*  *L. hajeki*  ***L. hatcheri***  *L. hellmichi*  ***L. irregularis***  *L. isabelae*  *L. jamesi*  *L. josei*  ***L. kingii***  ***L. koslowskyi***  ***L. kriegi***  ***L. laurenti***  ***L. lavillai***  ***L. lemniscatus***  ***L. leopardinus***  ***L. lineomaculatus***  *L. lorenzmuelleri*  *L. lutzae*  ***L. magellanicus***  ***L. major (= capillitas)***  ***L. melanops***  ***L. monticola***  *L. moradoensis*  ***L. morenoi***  ***L. multicolor***  ***L. multimaculatus***  *L. neuquensis*  *L. nigriceps*  *L. nigromaculatus*  ***L. nigroviridis***  ***L. nitidus***  *L. occipitalis*  ***L. olongasta***  ***L. ornatus***  ***L. pagaburoi***  *L. parvus*  *L. patriciaiturrae*  *L. paulinae*  ***L. petrophilus***  ***L. pictus***  *L. platei*  *L. pleopholis*  *L. poconchilensis*  *L. pseudoanomalus*  *L. pseudolemniscatus*  ***L. quilmes***  ***L. ramirezae***  *L. ramonensis*  *L. reichei*  *L. riojanus*  ***L. robertmertensi***  *L. robertoi*  *L. rosenmanni*  ***L. rothi***  ***L. ruibali***  ***L. sagei***  ***L. salinicola***  ***L. sarmientoi***  *L. saxatilis*^(3)^  ***L. scapularis***  *L. schroederi*  *L. scolaroi*  *L. scrocchii*  *L. silvanae*  *L. somuncurae*  *L. stolzmanni*  *L. tacnae*  ***L. tari***  ***L. tenuis***  *L. torresi*  ***L. uspallatensis***  *L. valdesianus*  *L. velosoi*  ***L. wiegmannii***  ***L. xanthoviridis***  ***L. yanalcu***  ***L. zapallarensis***  ***L. zullyi*** | 19  11  48  9  –  –  54  66  10  122  6  17  148  113  59  12  77  39  42  18  11  –  59  19  40  114  62  29  43  124  54  28  42  8  16  83  13  36  29  68  33  17  –  5^(2)^  18  49  29  22  16  57  38  53  14  32  12  14  193  35  57  20  11  54  8  33  59  30  12  21  18  43  14  22  204  61  14  24  28  14  13  16  26  34  137  49  27  11  12  14  17  9  28  8  16  –  28  35  43  91  19  17  71  –  19  43  29  10  49  31  5  –  19  181  9  20  39  60  25  26  –  89  47 | 61.1  93.6  50.1  72.2  54.6  –  85.3  69.6  54.0  93.7  45.2  92.3  74.7  56.3  70.8  59.4  63.2  93.8  94.3  87.0  45.8  –  91.3  76.0  63.1  69.2  61.6  82.9  55.5  58.6  72.4  85.2  79.9  51.4  55.3  94.3  80.7  47.5  89.3  74.4  50.7  55.8  –  91.0  64.5  63.0  57.2  84.0  79.7  93.1  64.6  88.8  61.6  97.0  54.9  55.8  49.9  88.5  60.2  95.2  83.5  59.6  99.3  84.3  64.1  59.2  86.7  81.5  71.0  61.5  88.9  53.6  76.9  96.6  70.0  66.9  68.5  53.6  67.8  91.9  53.3  93.7  65.3  54.2  75.4  59.4  56.5  49.9  65.6  51.8  88.8  59.2  58.7  –  63.7  74.3  93.6  58.7  87.1  74.5  86.2  58.0  67.2  56.4  60.9  94.4  75.4  85.7  88.6  –  93.4  57.2  56.1  64.5  88.8  51.9  55.9  82.3  –  92.6  78.5 | 60.2  80.2  47.8  68.0  49.7  –  84.1  61.8  50.3  88.4  45.4  86.2  72.9  56.3  57.5  61.3  63.6  90.9  82.3  82.5  50.4  –  92.3  75.0  61.0  60.6  56.1  77.8  61.3  58.4  64.3  79.4  78.6  48.9  55.5  89.3  76.1  44.1  84.7  70.5  49.5  57.1  –  89.9  58.9  64.1  50.4  73.8  68.1  87.5  58.9  84.2  59.6  99.7  51.5  55.0  47.8  85.9  56.3  80.4  70.0  59.8  83.0  78.7  60.3  59.0  77.8  74.8  65.0  61.2  86.6  50.6  63.8  86.5  61.0  64.9  63.8  52.7  62.5  86.8  52.6  90.3  64.9  52.9  70.0  47.9  63.0  44.0  63.9  51.3  88.2  50.9  51.8  –  58.7  66.2  92.9  56.3  84.9  67.9  84.8  55.1  58.4  60.8  56.7  86.2  72.9  83.9  83.9  –  90.8  56.7  58.0  63.1  89.1  46.8  55.9  82.3  –  82.8  69.2 | 27°19’S-27°47’S  23°30’S-24°26’S  17°00'S-21°35'S  30°35'S-34°20'S  30°05'S-30°55'S  37°28’S-38°50’S  46°38’S-47°10’S  23°55’S-28°30’S  22°08'S-23°03'S  34°30’S-37°45’S  27°34'S-27°50'S  49°10’S-49°30’S  33°11’S-33°21’S  32°00’S-49°00’S  26°20’S-27°50’S  23°24'S-28°38'S  41°00’S-44°05’S  36°00’S-38°50’S  43°00’S-44°03’S  34°55’S-38°48’S  23°45'S-33°28'S  42°08'S-43°00'S  31°22’S-39°24’S  36°50’S-39°27’S  38°38’S-38°42’S  22°37’S-23°55’S  34°08’S-35°03’S  27°19’S-33°00’S  36°40’S-41°45’S  28°28’S-42°55’S  29°06’S-29°10’S  50°30’S-50°40’S  22°55’S-23°45’S  28°45’S-28°55’S  32°46’S-32°55’S  44°00’S-50°00’S  22°41’S-22°44’S  30°30’S-36°35’S  47°33’S-47°55’S  39°00'S-43°20'S  29°09'S-42°45'S  33°25’S-33°35’S  35°14'S-38°20'S  37°55’S-39°30’S  21°19’S-22°20’S  47°42’S-48°02’S  23°28’S-23°30’S  23°55’S-24°11’S  26°14’S-26°26’S  17°00’S-20°55’S  34°53'S-36°51'S  43°00’S-51°40’S  27°11’S-29°18’S  34°00’S-42°04’S  28°10’S-30°12’S  24°37'S-25°26'S  30°26’S-39°40’S  33°15’S-33°21’S  41°50’S-51°30’S  29°49’S-30°13’S  22°53’S-23°53’S  51°00’S-53°57’S  27°03'S-27°54'S  36°26’S-43°00’S  33°11’S-34°11’S  33°42’S-33°45’S  38°47’S-41°06’S  21°40’S-24°20’S  35°00’S-41°01’S  37°47’S-37°51’S  24°00’S-28°42’S  23°50’S-28°30’S  32°58’S-34°04’S  28°15’S-36°20’S  27°02’S-33°11’S  28°38’S-31°14’S  21°35’S-24°15’S  26°44’S-27°30’S  28°29'S-32°27'S  26°14’S-26°26’S  22°27’S-22°28’S  41°20’S-43°50’S  35°27’S-43°23’S  25°00’S-31°38’S  18°12’S-18°12’S  18°00'S-18°25'S  28°15'S-31°45'S  29°56’S-32°10’S  24°43’S-26°50’S  24°20’S-27°20’S  33°24’S-33°30’S  20°10'S-20°30'S  29°00’S-32°00’S  27°57'S-28°21'S  29°47’S-30°28’S  26°27’S-28°42’S  38°50’S-41°25’S  32°27’S-32°55’S  39°01’S-40°17’S  27°00’S-32°07’S  52°00’S-52°15’S  30°09'S-33°09'S  23°00’S-32°00’S  33°16’S-36°37’S  46°49’S-46°52’S  22°57'S-25°01'S  47°17’S-47°23’S  40°44’S-40°50’S  21°29’S-22°50’S  17°37’S-18°04’S  49°12’S-49°16’S  32°01’S-41°44’S  22°22'S-22°24'S  32°32’S-32°40’S  33°47’S-33°56’S  26°23’S-27°23’S  17°17’S-40°50’S  37°00’S-44°00’S  24°13'S-24°21'S  30°00’S-33°00’S  46°42’S-47°13’S | 1200-1900  3060-4020  3000-4800  380-1975  0-20  1400-1700  610-1600  0-2000  2300-3200  900-2310  70-250  600-700  2100-3500  0-3000  0-500  700-2800  0-2000  1500-3000  600-900  1000-2300  690-820  817-986  0-2100  1500-2300  1500-2100  2200-3900  1520-1950  400-2000  0-800  800-3000  2500-3500  800-1100  2300-2450  3700-4200  2400-3200  0-1100  3200-3600  500-2100  1000-1300  0-200  0-1380  100-730  600-1200  500-1000  3500-3900  1000-1200  240-1785  3060-5000  2850-3672  3300-4700  900-2500  0-1340  800-2450  950-2000  800-1100  2800-4100  0-2100  1800-3000  780-1500  3200-3500  0-1200  0-1100  2500-4000  900-2070  1500-2500  2400-3600  740-1023  3600-4400  0-1000  1800-2200  3200-5100  0-250  500-3370  0-3153  0-250  900-1770  3500-4800  3000-4700  2905-3532  2850-3500  2200-2300  600-1400  0-1600  0-1050  4069-4400  700-1150  990-1700  50-800  1600-3000  2820-3300  2500-3000  580-1350  500-1000  690-2600  2400-3700  1960-4200  500-1903  2370-3000  931-1355  0-2050  0-900  700-1100  1000-2100  500-2590  850-920  4000-4900  1300-1600  1200-1400  3700-4300  2438-4080  280-1200  0-1800  2100-2500  1830-2200  1800-3500  0-750  0-2600  0-100  3730-4305  0-800  820-1400 |

1. Body size data taken from Verrastro et al. (2003).
2. Male body size taken from Avila et al. (2004) and female body size estimated from data shown in this paper and five available adult females.
3. Body size data personally provided by J.M. Cei.

**Supplementary References**

Avila, L. J., Morando, M., Perez, C. H. F. & Sites, J. W. 2004. Phylogenetic relationships of lizards of the *Liolaemus petrophilus* group (Squamata, Liolaemidae), with description of two new species from western Argentina. *Herpetologica* **60**: 187-203.

Verrastro, L., Veronese, L., Bujes, C. & Martins Dias Filho, M. 2003. A new species of *Liolaemus* from southern Brazil (Iguania: Tropiduridae). *Herpetologica* **59**: 105-118.
