# Supplementary material for: Predictable Variation of Range-Sizes across an Extreme Environmental Gradient in a Lizard Adaptive Radiation: Evolutionary and Ecological Inferences
Source: PLoS One. 2011 Dec 14;6(12):e28942. doi: 10.1371/journal.pone.0028942 (PMC3237565; doi:10.1371/journal.pone.0028942)
Supplement: Appendix S1 — This appendix contains references to the institutions that kindly provided permission to study their Liolaemus collections. Important part of the data used in this study comes from these museum collections (see Materials and methods). (DOCX) [file pone.0028942.s003.docx]

**Appendix S1**

This appendix contains references to the institutions that kindly provided permission to study their *Liolaemus* collections. Important part of the data used in this study comes from these museum collections (see Materials and methods).

Studied collections and their acronyms: Museo Nacional de Historia Natural de Chile (MNHNC); Zoological Museum, Universidad de Concepcion, Chile (MZUC); Museo de Historia Natural de Concepcion, Chile (MHNC); Universidad Nacional de Cuyo, Argentina (IBAUNC); Centro Nacional Patagonico, Argentina (CENPAT); Instituto Argentino de Investigaciones de las Zonas Aridas, Argentina (IADIZA); Universidad Nacional del Comahue, Argentina (UNComahue), Natural History Museum of London (NHML); Museum fuer Naturkunde of Berlin (ZMB); Natural History Museum of Vienna (NHMV); National Museum of Natural History of Paris (MNHNP); J.M. Cei Collection (JMC-DC); J.A. Scolaro Collection (JAS-DC).
